# Cognitive function of children with biliary atresia after primary living donor liver transplantation

**DOI:** 10.1186/s12887-024-04853-5

**Published:** 2024-06-01

**Authors:** Tingge Wang, Yan Hu, Zhanzhan Zhang, Xiaoke Dai, Mingman Zhang, Yan He, Yingcun Li

**Affiliations:** 1 Department of Child Health Care, Chongqing Key Laboratory of Pediatrics, Ministry of Education Key Laboratory of Child Development and Disorders, Children’s Hospital of Chongqing Medical University, National Clinical Research Center for Child Health and Disorders, Chongqing, 400014 China; 2 Department of Hepatobiliary Surgery, Chongqing Key Laboratory of Pediatrics, Ministry of Education Key Laboratory of Child Development and Disorders, Children’s Hospital of Chongqing Medical University, National Clinical Research Center for Child Health and Disorders, Chongqing, 400014 China; 3 Department of General Surgery, Beijing Children’s Hospital, Capital Medical University, National Center for Children’s Health, Beijing, 100045 China

**Keywords:** BA, pLDLT, Cognitive Development, Infant

## Abstract

**Background:**

The survival rate of children with biliary atresia (BA) after liver transplantation (LT) is significantly improved, and their quality of life has attracted much attention.This study aimed to investigate the cognition and its influencing factors in children with BA after primary living donor LT (BA-pLDLT) during infancy.

**Methods:**

Children with BA were recruited 6 months after pLDLT at Children’s Hospital of Chongqing Medical University (2018–2022). Demographic and clinical data were collected from the health information system. Cognition was assessed using the Chinese version of the Griffiths Mental Development scale (GMDS-C). Multivariate linear regression were used to analyze the influencing factors of their cognitive function.

**Results:**

In total, 57 children with BA-pLDLT, aged 5.00(3.90–9.30) months at transplantation and 25.00(14.00-60.80) months at evaluation were included. The general developmental quotient (89.02 ± 12.07) and motor, language, eye-hand coordination, performance, and practical reasoning quotients of these children were significantly lower than the normative mean values of GMDS-C(*P* < 0.05). Of the 57 children, 16 (28.07%) had borderline developmental delay (DQ between 70 and 84), 3 (5.26%) had developmental delay (DQ < 70), and 11(19.29%) had language delay. Reoperation for biliary or vascular complications after pLDLT was a risk factor for decreased general development quotient and motor quotient and lower Z_W_ at assessment was associated with decline motor quotient.

**Conclusion:**

Children with BA-pLDLT have varying degrees of developmental delays in early life. Reoperation and nutritional deficiencies had adverse effects on cognitive development.

## Introduction

Biliary atresia (BA), the most common, serious disease in infants, leads to liver cirrhosis and failure. Most patients die within 2 years of age in the absence of surgery. China has a significantly higher incidence of BA than in Europe and the United States, with 1/9200 cases reported in Shanghai in 2020 [1]. Liver transplantation (LT) is the only curative treatment for BA. Although early Kasai portoenterostomy (KP) may improve cholestasis to some extent, approximately 67% of the patients still require LT before adulthood [2]. And it is reported that the long-term survival rate with salvage LT is not as good as that of primary living donor liver transplantation (pLDLT) for BA patients [3, 4]. Therefore, an increasing number of children in China with BA, aged below 1 year, are undergoing primary living donor liver transplantation (pLDLT) as a radical treatment.

With the development of LT techniques and immunosuppressants, the annual survival rate of patients after LT is increasing yearly [5]. Therefore, clinicians have started focusing on long-term quality of life, including neurocognitive development [6, 7]. In our previous study, we found that the head circumference of children with BA, even before transplantation, was significantly lower than that of normal children, and despite catch-up growth of the head circumference after early LT, the cognitive development level of some children still lagged behind [8]. Impaired language and motor function before LT has been reported in children with BA [9], suggesting that the disease itself may cause impairment. In addition, intelligence and quality of life of children after LT have been reported [6, 7, 10]. Rodijk et al. analyzed 36 school-aged children who underwent LT for BA and found that their intelligence quotient (IQ) was significantly lower than that of the normal population, especially in the area of motor skills [11]. In 2019, Zhu et al. reported that 95 Chinese children aged 2.5–6.9 years, after one year of LT, experienced a decline in full-scale intelligence quotient(FSIQ) and verbal comprehension index(VCI) [12].

Although the cognitive function of children after LT has been reported in recent studies, the children who were included in these studies had other diseases besides BA such as genetic metabolic diseases, acute liver disease, and other conditions [6, 10, 12–14]. Hence, the cognitive function of these populations does not accurately represent the neurocognitive developmental status of children with BA after LT. Moreover, most of the reports showed that children with BA received sequential treatment with KP and LT, and the age at LT ranged from 0.4 to 11.5 years [11, 15]. However, poor resolution of jaundice and cholangitis are not rare in children after KP, and persistent cholestasis may have adverse effects on the developing brain. Therefore, the cognitive function of these patients may be different from that of children after pLDLT. In addition, most of the children in the previous studies received donation after cardiac death, which makes it difficult to determine the effect of pLDLT surgery on neurodevelopment [6, 10, 14].

Herein, we aimed to determine the level of neurocognitive development and the factors influencing it in children with BA who underwent pLDLT (BA-pLDLT) during infancy.

## Materials and methods

### Participants

The participants were recruited from 170 children who underwent LDLT at the Children’s Hospital of Chongqing Medical University from 2018 to 2022. The inclusion criteria were as follows: at least 6 months after BA-pLDLT, stable condition in the past 1 month, volunteer to participate and informed consent granted by parents/legal guardians. Children who had other congenital disorders (including genetic or metabolic diseases) or postoperative diseases (including encephalitis and traumatic brain injury) that seriously affected cognitive development or re-transplantation were excluded.

### Participants’ information

Demographic information including sex, chronological age (CA), age at LT, age at diagnosis (days) and educational level of the caregiver was collected. Clinical data related to the disease, including preoperative blood ammonia level, lactic acid, liver function, preoperative end-stage liver disease (PELD) score, total hospital stay during LT, number of days in the intensive care unit (ICU) after LT, duration of ventilator use after LT, and reoperation due to complications, were obtained from the hospital’s health information system. Anthropometric measurements (weight, length, and head circumference) were obtained by trained pediatric nurses and interpreted by trained pediatricians. The Z values for weight-for-age (Z_W_), length-for-age (Z_L_), and head circumference-for-age (Z_HC_) were calculated using the WHO standards software (WHO Anthro).

### Neurocognitive developmental assessment

The Chinese version of the Griffiths Mental Development Scale (GMDS-C) was used to evaluate cognitive function. The GMDS-C is applicable to children ages 0–8 years and includes a 0 ~ 2year scale (A-locomotor, B-personal-social, C-language, D-eye‐hand co‐ordination, E-performance) and 2–8year scale (by adding F-practical reasoning) [16]. The assessment was completed by certified evaluators and have a GMDS-C assessor examiner conduct regular quality monitoring. According to the scoring principles of the GMDS-C, the raw scores of the six domains were calculated and converted into developmental age (DA) according to the Chinese norms; the developmental quotient(DQ) was calculated as The DQ = DA/CA. The DQ of the six domains were termed as A-Q, B-Q, C-Q, D-Q, E-Q, F-Q, and the general DQ was expressed as G-Q. The DQ < 70 denoted developmental delay, 70–84, borderline delay, 85–114, normal development, 115–130, normal-to-high development, and > 130 denoted advanced development.

### Statistical analysis

The sample size was calculated using statistical software PASS 15 Power Analysis and Sample Size Software (2017; NCSS, LLC. Kaysville, Utah, USA, ncss.com/software/pass). It has been reported that the incidence of developmental delay after LT in children is approximately 4–27% [14], while in the normal population, it is 1–2%. Based on these proportions, double-sided α = 0.05, and 90% confidence level, the sample size was determined to be 36.

IBM SPSS Statistics for Windows, version 25.0 (IBM Corp., Armonk, NY, USA) was used for statistical analyses. After normality testing, qualitative data are described as number (percentage), and quantitative data, as median (ranges) or mean ± standard deviation (X ± S). One-sample t-test was used to compare the DQ between children with BA-pLDLT and the normative mean value of GMDS-C. Multivariate linear regression analysis was used to identify the factors influencing DQ in children with BA-pLDLT. Statistical significance was set at *P* < 0.05.

## Results

### Participants’ information

Among the 57 children with BA-pLDLT included in this study, 33 were male, and the median age was 5.00 (3.90, 9.30) months at LT. The age at cognitive assessment was 25(14.00,60.80) months. The interval between LT and cognitive assessment was 20.90(9.90,53.60) months. Immunosuppressants [ tacrolimus + mycophenolate mofetil (MMF) + steroids] were routinely used for 1 to 6 months after surgery and the steroids/ MMF were stopped gradually. At the time of evaluation, 38 of the 57 children were maintained on tacrolimus monotherapy, and 19 were maintained on combined therapy tacrolimus + MMF. Of the 57 children, 15 underwent reoperation within 1 ~ 2 years due to biliary or vascular complications (biliary anastomotic stenosis, portal vein stenosis and hepatic vein stenosis). (Table 1)

**Table 1:** General demographic information and clinical data (*N* = 57)

Items		Items	
Age at diagnosis (days)	70.00(34.00-160.00)	Total hospital stay during LT (day)	33.5(21, 86)
Age at LT (month)	5.00 (3.90–9.30)	Days in ICU after LT (day)	5(2, 49)
Z_W_ at LT	-1.07 ± 1.08	Duration of ventilator use after LT (day)	2 (1, 30)
Age at assessment (month)	25.00(14.00-60.80)	PELD score at LT	17(3, 40)
Time from transplant to assessment (month)	20.15(9.90–53.60)	The preoperative biochemical tests	
Z_W_ at assessment	-0.02 ± 0.99	Blood ammonia (umol/L)	50.38 ± 19.28
Z_L_ at assessment	-0.84 ± 1.22	Lactic acid ( mmol/L)	3.27(1.17,47.70)
Z_HC_ at assessment	-0.16 ± 1.06	TBA ( umol/L)	183.70(33.80, 488.50)
Gender		Total bilirubin ( umol/L)	327.08 ± 134.91
male	33 (57.89%)	ALT ( U/L)	278.75(72.60-807.30)
female	24 (42.11%)	AST ( U/L)	433.30(133.10–1215.00)
Family residence		GGT ( U/L)	356.00(38.00-2557.00)
city	26(45.61%)	Donor–recipient ABO matching	
county	15(26.32%)	Identical	29(50.88%)
town	16(28.07%)	Nonidentical	28(49.12%)
Educational level of the caregiver		Re-operation for biliary/vascular complications	
Junior high school and below	38(66.67%)	yes	15(26.32%)
senior high school	9 (15.79%)	no	42(73.68%)
College/Bachelor degree or above	10 (17.54%)	Long-term immunosuppressant regimen	
		Tacrolimus	38(66.67%)
		Tacrolimus + MMF	19(33.33%)

Note: Measurements are expressed as mean ± standard deviation or median (range), and counts are expressed as frequency (n) and percentage (%)

BA-pLDLT: the children with biliary atresia after primary living donor liver transplantation; LT: liver transplantation; ICU: intensive care unit; PELD: preoperative end-stage liver disease; Zw: Weight-for-age Z-score; Z_L_: Length-for-age Z-score; Z_HC_: Head circumference-for-age Z-score; ALT: Alanine aminotransferase; AST: aspartate aminotransferas; GGT: γ-glutamyl transpeptidase; TBA: total bile acids; MMF: mycophenolate mofetil

### Cognitive function in children with BA-pLDLT

The G-Q of the 57 children with BA-pLDLT was 89.02 ± 12.07. The G-Q and the DQ of the locomotor, language, eye-hand coordination, performance, and practical reasoning domains were significantly lower in the patient group than in the normative mean value of GMDS-C (*P* < 0.05), but there was no difference in the DQ of the personal-social domain. (Table 2). To analyze the impact of the duration since LT on the cognitive level, children with BA-pLDLT were divided into < 2 years after LT group(*N* = 32) and ≥ 2 years after LT group (*N* = 25). The results shew that only the A-Q score of the group ≥ 2 years after LT was significantly lower than that of the group < 2 years after LT (94.46 ± 10.91 vs. 83.00 ± 14.34, *P* = 0.001). (Table 2)

**Table 2:** Cognition of children with BA-pLDLT

	G-Q	A-Q	B-Q	C-Q	D-Q	E-Q	F-Q^a^
Comparison with the norm population (normative mean value = 100)							
BA-pLDLT^b^ (*n* = 57)	89.02 ± 12.07	89.44 ± 13.67	95.97 ± 19.99	87.59 ± 20.50	91.76 ± 13.21	89.02 ± 12.07	83.87 ± 18.89
***t***	-6.868	-5.834	-1.521	-4.571	-4.706	-4.706	-3.623
***p***	< 0.001	< 0.001	0.134	< 0.001	< 0.001	< 0.001	0.002
Comparison between within and after 2 years of LT							
< 2 years after LT (*n* = 32)	91.42 ± 12.35	94.46 ± 10.91	92.47 ± 19.57	88.65 ± 23.89	92.75 ± 12.37	89.94 ± 10.61	
≥ 2 years after LT(*n* = 25)	85.95 ± 11.18	83.00 ± 14.34	100.45 ± 19.99	86.23 ± 15.49	90.51 ± 14.38	90.38 ± 15.12	
***t***	1.731	3.427	-1.509	0.439	0.632	-0.130	
***p***	0.089	0.001	0.136	0.663	0.530	0.897	

Note: a: Because the evaluation of F domain is suitable for children over 2 years old, a total of 18 children were included in this group. b: The cognitive level of BA-pLDLT children was compared with the norm population value of 100

BA-pLDLT: the children with biliary atresia after primary living donor liver transplantation during infancy; GMDS-C: Griffiths mental development scales (Chinese version); G-Q: the general developmental quotient; A-Q: Locomotor developmental quotient; B-Q: Personal-social developmental quotient; C-Q: Language developmental quotient; D-Q:Eye-hand co-ordination developmental quotient; E-Q: Performance developmental quotient; F-Q: Practical Reasoning developmental quotient;

Among the 57 children, 3(5.26%) had developmental delay and 16 (28.07%) had borderline developmental delay. Language impairment was the most prominent impairment, with an average DQ of 87.59 ± 20.50. Eleven (19.29%) patients had a delay in language acquisition and 17 (29.82%) had borderline delay. Of the 57cases, 30 were children over 2 years old, but only 18 completed the assessment in the F domain; significant impairment was found in practical reasoning ability after 2 years of age. (Table 3)

**Table 3:** Distribution of BA-pLDLT developmental quotient

DQ	developmental delay *n*(%)	Borderline *n*(%)	Normal *n*(%)	normal high values *n*(%)	developmental advances *n*(%)
G-Q(*n* = 57)	3(5.26%)	16(28.07%)	37(64.91%)	1(1.76%)	0(0%)
A-Q(*n* = 57)	3(5.26%)	18(31.58%)	34(59.65%)	2(3.51%)	0(0%)
B-Q(*n* = 57)	4(7.02%)	12(21.05%)	34(59.65%)	5(8.77%)	2(3.51%)
C-Q(*n* = 57)	11(19.29%)	17(29.82%)	24(42.11%)	4(7.02%)	1(1.76%)
D-Q(*n* = 57)	4(7.02%)	11 (19.29%)	41(71.93%)	1(1.76%)	0(0%)
E-Q(*n* = 57)	3(5.26%)	15(26.32%)	37(64.91%)	2(3.51%)	0(0%)
F-Q(*n* = 18)	3(16.67%)	8(44.44%)	6(33.33%)	0(0%)	1(5.56%)

Note: DQ < 70 was considered as developmental delay, 70–84 was borderline, 85–114 was normal, 115–130 was normal high values, > 130 was developmental advances. G-Q: the general developmental quotient; A-Q: Locomotor developmental quotient; B-Q: Personal-social developmental quotient; C-Q: Language developmental quotient; D-Q:Eye-hand co-ordination developmental quotient; E-Q: Performance developmental quotient; F-Q: Practical Reasoning developmental quotient

### Factors influencing cognitive function in children with BA-pLDLT

Univariate linear regression analysis was performed to identify the factors affecting cognitive function in children with BA-pLDLT. The results revealed that Z_W_ at assessment (B = 3.570, *P* = 0.037) and reoperation due to biliary or vascular complications after pLDLT (B=-8.034, *P* = 0.027) affected the G-Q. Additionally, age at assessment (B=-0.394, *P* = 0.026), time from transplant to assessment (B=-0.405, *P* = 0.032), Z_W_ at the time of assessment (B = 5.999, *P* = 0.001), total hospital stay during LT (B=-0.346, *P* = 0.017) and reoperation for biliary or vascular complications (B=-13.852, *P* = 0.001) affected A-Q. (Table 4)

**Table 4:** Univariate linear regression analysis of factors influencing cognition in children with BA-pLDLT

Variables	G-Q	A-Q	C-Q	D-Q	E-Q	F-Q
Age at LT (month)	B=-2.495 *P* = 0.060	B=-2.839 *P* = 0.059	B=-1.284 *P* = 0.574	B=-1.938 *P* = 0.185	B=-1.450 *P* = 0.303	B=-2.287 *P* = 0.493
Z_W_ at LT	B = 1.142 *P* = 0.451	B = 3.117 *P* = 0.066	B=-0.627 *P* = 0.808	B = 0.444 *P* = 0.790	B=-0.723 *P* = 0.650	B=-1.825 *P* = 0.720
Age at assessment (month)	B=-0.180 *P* = 0.255	B=-0.394 *P* = 0.026	B = 0.026 *P* = 0.923	B=-0.094 *P* = 0.589	B = 0.017 *P* = 0.918	B = 0.135 *P* = 0.780
Time from transplant to assessment (month)	B=-0.166 *P* = 0.327	B=-0.405 *P* = 0.032	B = 0.050 *P* = 0.862	B=-0.077 *P* = 0.681	B = 0.043 *P* = 0.810	B = 0.215 *P* = 0.681
Z_W_ at assessment	B = 3.570 *P* = 0.037	B = 5.999 *P* = 0.001	B = 5.718 *P* = 0.047	B = 2.360 *P* = 0.211	B = 0.783 *P* = 0.667	B=-3.580 *P* = 0.600
Z_L_ at assessment	B = 1.336 *P* = 0.344	B = 2.709 *P* = 0.086	B = 2.665 *P* = 0.261	B = 1.097 *P* = 0.476	B = 1.310 *P* = 0.374	B=-1.023 *P* = 0.854
The PELD score at LT	B= -0.204 *P* = 0.259	B=-0.369 *P* = 0.069	B=-0.168 *P* = 0.588	B=-0.105 *P* = 0.598	B=-0.259 *P* = 0.172	B=-0.241 *P* = 0.637
Total hospital stay during LT (day)	B=-0.069 *P* = 0.599	B=-0.346 *P* = 0.017	B=-0.040 *P* = 0.859	B=-0.001 *P* = 0.996	B = 0.120 *P* = 0.382	B = 0.128 *P* = 0.662
Re-operation for biliary/vascular complications	B=-8.034 *P* = 0.027	B=-13.852 *P* = 0.001	B=-7.889 *P* = 0.209	B=-2.053 *P* = 0.610	B = 1.867 *P* = 0.628	B=-0.681 *P* = 0.945
Pre-blood ammonia	B=-0.055 *P* = 0.539	B=-0.110 *P* = 0.263	B=-0.147 *P* = 0.333	B=-0.077 *P* = 0.426	B=-0.007 *P* = 0.939	B = 0.187 *P* = 0.446
Pre-TBA	B = 0.024 *P* = 0.105	B = 0.025 *P* = 0.166	B = 0.051 *P* = 0.053	B = 0.018 *P* = 0.226	B = 0.006 *P* = 0.692	B=-0.004 *P* = 0.917
Educational level of the caregiver	B = 6.968 *P* = 0.091	B = 5.499 *P* = 0.243	B = 10.983 *P* = 0.115	B = 3.548 *P* = 0.444	B = 4.530 *P* = 0.303	B = 5.314 *P* = 0.604
Family residence	B=-1.412 *P* = 0.715	B=-5.570 *P* = 0.197	B=-3.725 *P* = 0.571	B=-2.171 *P* = 0.601	B=-0.582 *P* = 0.884	B = 13.766 *P* = 0.138

Note: G-Q: the general developmental quotient; A-Q: Locomotor developmental quotient; B-Q: Personal-social developmental quotient; C-Q: Language developmental quotient; D-Q: Eye-hand co-ordination developmental quotient; E-Q: Performance developmental quotient; F-Q: Practical Reasoning developmental quotient

Factors identified in univariate linear regression analysis were included in the multivariate linear regression analysis, and the results showed that reoperation for biliary or vascular complications after transplantation was the risk factor for the children lagging behind in terms of the G-Q (B=-8.156, *P* = 0.031, R^2^ = 0.071) and A-Q (B=-11.019, *P* = 0.005, R2 = 0.270). Lower Z_W_ at assessment (B = 4.667, *P* = 0.010, R^2^ = 0.270) was associated with decline motor developmental quotient. (Table 5)

**Table 5:** Multivariate linear regression analysis of factors influencing cognitive function in children with BA-pLDLT

	Variables	B	β	t	*P*	B (95%CI)	*R* ^2^
G-Q	Re-operation for biliary/vascular complications	-8.156	-0.297	-2.225	0.031	(-15.515, -0.798)	0.071
A-Q	Re-operation for biliary/vascular complications	-11.019	-0.357	-2.906	0.005	(-18.634, -3.403)	0.270
A-Q	Z_W_ at assessment	4.667	0.331	2.695	0.010	(1.188, 8.145)	0.270

Note: G-Q: the general developmental quotient; A-Q: Locomotor developmental quotient

### Comparison of clinical features between reoperation and non-reoperation group

From the above, reoperation seems to be a risk factor affecting the postoperative cognition of children. It is very important to reduce the probability of reoperation. Therefore, we further analyzed the clinical characteristics of children undergoing reoperation. The children were divided into two groups, no-reoperation group and reoperation group. Their clinical data were reanalyzed and it was observed that there was a significant difference in age at LT, age at assessment between the two groups, the reoperation group being older. Furthermore, the PELD score at LT and total hospital stay during LT were significantly different between the two groups. (Table 6)

**Table 6:** Comparison of clinical features between reoperation and non-reoperation group

Items	No Re-operation group *N* = 42	Re-operation group *N* = 15	t	*P*
Age at LT (month)	5.13 ± 0.95	6.30 ± 1.46	-3.531	0.004
Age at assessment (month)	25.38 ± 7.76	37.00 ± 11.75	-4.318	0.002
total hospital stay during LT (day)	31.76 ± 7.86	47.13 ± 15.96	-4.854	< 0.001
the PELD score at LT	16.77 ± 8.06	23.53 ± 9.77	-2.638	0.015

## Discussion

To our knowledge, this is the first study to specifically focus on BA-pLDLT children aiming to clarify their cognitive development in early life. We found that cognitive impairment, particularly language, occurred in the early postoperative period in children with BA-pLDLT and that reoperation for biliary or vascular complications increased the risk of deficits in general intelligence and motor developmental quotient, the poor nutritional status at the time of assessment is also a risk factor for delayed motor development.

Although our findings are consistent with previous studies showing cognitive impairment in children after liver transplantation [11, 14]. However, in previous studies the children were older at the time of transplantation and assessment [10, 14, 17]. 0 ~ 2 years is a critical period for brain development, and early adverse experiences have a negative impact on cognitive function that may extend even to adolescence and adulthood. Our study focuses on the early postoperative period (median 20.15 months) in children with BA-pLDLT during infancy, suggesting that cognitive impairment occurs earlier in such children.

The results showed that while the G-Q of preschool children with BA-pLDLT fell within the normal range (89.02 ± 12.07), the rates of developmental delay and borderline status were 5.26% and 28.07%, respectively. This means that early after surgery, children with BA-pLDLT experience comprehensive cognitive impairment necessitating cognitive-behavioral intervention early after transplantation. Zhu et al. found that the IQ of children decreased with increasing age at LT [12], suggesting that a prolonged waiting time for LT in children with chronic liver disease has an irreversible impact on cognitive development. Recently, a longitudinal study reported that at 6 months after LDLT, although the children’s developmental milestone was delayed compared to that of the normal pediatric population, it had significantly improved compared to that before surgery [18]. Therefore, impaired postoperative cognitive development in children with BA may gradually improve over time, indicating the benefit of neurodevelopmental plasticity in early life.

The period of the first 1000 days of life is a critical time for motor and language development. Children with BA prior to LT experience delayed development of gross motor function and language, during infancy [9]. In our study, we found that children who underwent BA-pLDLT in infancy also exhibited impairments in multiple cognitive domains during preschool age, especially in language acquisition. Although the highest prevalence of language delay is reported at ages 2 ~ 3 years, in our study, 19.29% (11/57) of the children with BA-pLDLT showed a delay and 29.82%(17/57) had borderline delay in this domain, which is higher than the incidence of language delay of 15% reported in previous studies [19]. This finding is consistent with those of Zhu et al. [12] and Ostensen et al [20]. Early language delays in children not only impact their language comprehension and expression but also have long-term effects on their learning, communication, and social relationships [21]. Early language development monitoring and interventions are important for children with BA-pLDLT in order to improve their cognition later in life.

Both environmental and genetic factors influence cognitive development. Various factors have been reported to affect cognitive function in children after LT. However, the results of different studies have been inconsistent, where factors such as parental educational level [13, 14], use of immunosuppressive drugs [22], preoperative growth retardation [13, 22], days in the ICU after LT [14], age at the time of LT [14] and preoperative blood ammonia and bilirubin levels [18, 22] have been implicated. In contrast to the above studies, we found that the risk factors associated with decreased G-Q and A-Q scores were reoperation for biliary or vascular complications and Z_W_ at assessment were associated with A-Q.

Nutrition in early life is crucial for physical growth and cognitive development. It has been reported that children after LT are prone to low body weight [23], obesity or metabolic syndrome in adulthood [24–28]. The findings of this study suggests that appropriate nutrition to promote catch-up growth after LT is essential for their cognitive development.

On the other hand, previous studies have shown that early and repeated exposure to anesthesia may have negative neurodevelopmental impact and be an independent risk factor for learning disabilities [29]. Children who are repeatedly exposed to anesthesia before the age of 3 years may experience decreased processing speed and fine motor abilities as well as increased problems related to executive function, behavior, and reading [30]. Hence, we speculate that repeated anesthesia for reoperation after LT in early life may have adverse effects on the immature brains. In addition, the prolonged hospital stay caused by reoperation, the anxiety of parents will limit the activities of children and ultimately affect their motor ability recovery.

Next, we further analyzed the clinical characteristics of children undergoing reoperation. We found that 15 children who underwent reoperation were older at transplantation and evaluation, had a higher PELD score, and a longer total hospital stay during LT period than those who did not undergo reoperation. Therefore, in order to reduce the aggravation of children’s condition caused by waiting and damage to later cognitive function, we suggested that liver transplantation should be performed at around 5 months of age or even earlier in infant with BA who chose primary liver transplantation in addition to routine treatment such as relieving jaundice.

Although our study described interesting results, it still had some limitations. First, this was a single-center cross-sectional study, it is unclear whether early cognitive-behavioral interventions can reverse cognition in children with BA-pLDLT and need to be confirmed in the future. Second, due to the small number of patients with KP-LT in our center, we did not compare the cognitive level of BA children who underwent KP-LT with those who underwnet pLDLT, so it cannot conclude which regimen is better at present.

## Conclusions

To conclude, we found that children with BA-pLDLT had a lower cognitive level than the norm population in early life, reoperation and lower body weight at assessment were independent risk factors for their postoperative cognitive delay. Shortening the waiting time of BA-pLDLT may reduce the risk of reoperation and thus alleviate cognitive impairment. Early postoperative nutritional support may be beneficial to the cognitive development of patients.

## Data Availability

The datasets used and/or analysed during the current study available from the corresponding author on reasonable request.

## Abbreviations

BA: Biliary atresia
CA: Chronological age
DA: Developmental age
GQ: General developmental quotient
GMDS-C: Griffiths Mental Development Scale (Chinese version)
FSIQ: Full-scale intelligence quotient
IQ: Intelligence quotient
PRI: Perceptual reasoning index
PSI: Processing speed index
KP: Kasai portoenterostomy
pLDLT: Primary living donor liver transplantation
MMF: Mycophenolate mofetil
PELD: Preoperative end-stage liver disease
Z_W_: Z score for weight-for-age
Z_L_: Z score for length-for-age
Z_HC_: Z score for head circumference-for-age
